# Supplementary material for: Needs Assessment of Southeastern United States Vector Control Agencies: Capacity Improvement Is Greatly Needed to Prevent the Next Vector-Borne Disease Outbreak
Source: Trop Med Infect Dis. 2022 May 13;7(5):73. doi: 10.3390/tropicalmed7050073 (PMC9143300; doi:10.3390/tropicalmed7050073)
Supplement: Supplementary file 1 [file tropicalmed-07-00073-s001.zip › tropicalmed-1710357-supplementary.pdf]

Tropical Medicine and Infectious Disease

MDPI

Supplementary Materials: Mosquito and Vector Control Agency Capacity and Needs Assessment for Emerging Vector-Borne Disease Threats in the Southeastern United States

Kyndall Braumuller[1], Jennifer R Gordon[2], Danielle Johnson[1], Josie Morrissey[1], Kaci McCoy[3], Rhoel R Dinglasan[3] and Melissa S Nolan[1]*

Thank you for your time and interest! This questionnaire is confidential (we will not ask any personal identifying information), and your participation is voluntary. The questionnaire should take less than 10 minutes to complete. Your answers will help us to inform policy makers as to the needs of local mosquito and vector control agencies to strengthen the overall vector-borne disease management and capacities in the southeastern United States.

Vector Control Capacity, Barriers and Needs Assessment Questionnaire

1. In what state do you operate? ___________________________
2. Do you work for a county, city, regional or state mosquito/vector control district/agency? _______________
3. Is your organization a part of the local public health agency or separate? Yes (apart) No (separate)
4. What is your job title? ____
5. What is your educational or training background? ____
6. What is the estimated population size of the residents you serve?
   0-10,000 10,001-100,000 100,001-1,000,000 1 million+
7. What vector borne diseases have you seen in your county? *(Circle all that apply)*
   West Nile virus Eastern equine encephalitis LaCrosse encephalitis
   St. Louis Encephalitis Spotted fever group rickettsiosis Lyme disease
   Murine typhus Chagas disease Other: _______________________
8. What pest(s) does(do) your organization control for: *(Circle all that apply)*
   Mosquitos Ticks Kissing bugs Bed bugs Sandflies Fleas Other pests (spiders, rats, etc.)
9. What time of year do you conduct surveillance? Summer All year long Other: ________________________
10. What types of surveillance do you conduct? Vector collections Pathogen testing Other: ______________
11. What treatment thresholds do you abide by? *(Circle all that apply)*

Presence of nuisance insects Positive mosquito pool Positive human case Positive avian/equine case Pre-determined schedule Other: ______________________

12. Which adulticides does your organization utilize? *(Circle all that apply)*

Malathion Permethrin Other:

____

13. What larvicides does your organization utilize? *(Circle all that apply)* Biological control Growth regulators Contact insecticides Stomach insecticides Other:

____

14. With what frequency does your organization apply insecticides?

Everyday Twice a week Weekly Other:

____

15. In what manner(s) do you apply insecticides? *(Circle all that apply)* Organization-owned truck Organization-owned aerial Contractor Other: ____

16. How many trucks equipped for spraying insecticides does your organization own? __________________

17. How many airplanes and/or helicopters equipped for spraying insecticides does your organization own? ___

18. Does your organization conduct their own vector speciation? Yes or No

If yes, please describe vector type (mosquito, ticks, etc) **and** process (subsampling, all collected vectors, etc.): __________________

19. What type of mosquitos have you seen in your region? *(Circle all that apply)* *Ae. aegypti* *Ae. albopictus* *Ae. triseriatus* *Cx. pipiens* *Cx. restuans* *Cx. nigripalpus* *Cx. salinarius* *Anopheles quadrimaculatus* *Culiseta melanura*

20. What type of ticks have you seen in your region? *(Circle all that apply)*

Amblyomma americanum *Dermacentor variabilis* *Ixodes scapularis* *Amblyomma maculatum* *Rhipicephalus sanguineus* *Haemaphysalis longicornis* Other: ____

21. How do you perform disease testing? *(Circle all that apply)*

Send to an outside laboratory In-house Don't perform any testing Other: ____________________________

22. Is there someone in your agency that performs outreach and education to the community you serve? Yes No If yes, what type of outreach/education is performed? *(Circle all that apply)* School-based programs Health-fairs Media Website/online presence Mobile units Other: ____________________________________

23. Is there someone in your organization that performs GIS or the mapping services? Yes No

24. Is there someone in your organization that performs insecticide resistance testing? Yes No If yes, how do you conduct resistance testing? Field cage tests Bottle bioassays Other: ________________

25. Is your agency prepared for a natural disaster-related vector-borne disease threat? Yes No

If yes, please elaborate: ____

26. What are the additional testing or control methods that you would like to use in your region, but do not have the resources for? Please elaborate. (Examples: additional chemical or biological control methods / pathogen testing / insecticide resistance testing)

____

____

27. In the event of a natural disaster, how timely are funds and/or resources normally available for response?

Within 24 hours Within 72 hours Not at all Other:

____

28. Are your testing and/or application equipment up-to-date and reliable? Yes No

29. Have faulty equipment ever halted a necessary control or surveillance activities? Yes No

If yes, please elaborate: ____

30. What gaps or needs do you see in your vector control program as the most urgent for vector prevention and control? Resources Personnel Funds Training Other: ____

31. What type of training does your organization most need? Vector identification Testing Other: ________________

32. What training dissemination style would be optimal for your organization? One day regional Online webinar Out of town workshop during non-peak season Working with an extension agent to hold on-site training

Other: ____

33. Thank you for your participation! Are there any final thoughts you would like to share with us?

____

____

__
